# Dementia resources for eating, activity, and meaningful inclusion (DREAM) toolkit co-development: process, output, and lessons learned

**DOI:** 10.1186/s40900-023-00497-4

**Published:** 2023-09-30

**Authors:** Laura E. Middleton, Shannon Freeman, Chelsea Pelletier, Kayla Regan, Rachael Donnelly, Kelly Skinner, Cindy Wei, Emma Rossnagel, Huda Jamal Nasir, Tracie Albisser, Fatim Ajwani, Sana Aziz, William Heibein, Ann Holmes, Carole Johannesson, Isabella Romano, Louisa Sanchez, Alexandra Butler, Amanda Doggett, M. Claire Buchan, Heather Keller

**Affiliations:** 1 University of Waterloo, 200 University Ave. W., Waterloo, ON N2L3G1 Canada; 2 Schlegel-UW Research Institute for Aging, 250 Laurelwood Dr, Waterloo, ON N2J 0E2 Canada; 3 University of Northern British Columbia, 3333 University Way, Prince George, BC V2N 4Z9 Canada; 4 Active Health Solutions, 150 - 556 North Nechako Road, Prince George, BC V2K 1A1 Canada; 5 University Health Network, 550 University Ave, Toronto, ON M5G 2A2 Canada; 6 Alzheimer Society of B.C., 828 W 8Th Ave Suite 300, Vancouver, BC V5Z 1E2 Canada

**Keywords:** Dementia, Care partner, Co-development, Patient and public involvement, Physical activity, Nutrition, Wellbeing, Training, Education, Toolkit

## Abstract

Promoting wellbeing of persons with dementia and their families is a priority of research and practice. Engaging diverse partners, including persons with dementia and their families, to co-develop interventions promotes relevant and impactful solutions. We describe the process, output, and lessons learned from the dementia resources for eating, activity, and meaningful inclusion (DREAM) project, which co-developed tools/resources with persons with dementia, care partners, community service providers, health care professionals, and researchers with the aim of increasing supports for physical activity, healthy eating, and wellbeing of persons with dementia. Our process included: (1) Engaging and maintaining the DREAM Steering Team; (2) Setting and navigating ways of engagement; (3) Selecting the priority audience and content; (4) Drafting the toolkit; (5) Iterative co-development of tools and resources; (6) Usability testing; and (7) Implementation and evaluation. In virtual meetings, the DREAM Steering Team confirmed the toolkit audiences (primary: community service providers; secondary: persons with dementia and care partners) and identified and evolved content areas. An environmental scan identified few existing, high-quality resources aligned with content areas. The Steering Team, additional multi-perspective partners, and external contractors iteratively co-developed new tools/resources to meet gaps over a 4-month virtual process that included virtual meetings, email exchange of documents and feedback, and one-on-one calls by telephone or email. The final DREAM toolkit includes a website with seven learning modules (on the diversity of dementia, rights and inclusion of persons living with dementia, physical activity, healthy eating, dementia-inclusive practices), a learning manual, six videos, nine handouts, and four wallet cards (www.dementiawellness.ca). Our co-development participants rated the process highly in relation to the principles and enablers of authentic partnership even though all engagement was virtual. Through use of the co-developed DREAM toolkit, we anticipate community service providers will gain the knowledge and confidence needed to provide dementia-inclusive wellness programs and services that benefit persons with dementia and their families.

**Supplementary Information:**

The online version contains supplementary material available at 10.1186/s40900-023-00497-4.

## Introduction

The number of persons with dementia is projected to more than double between 2020 and 2050, reaching over 115 million people globally [1]. Pharmaceutical treatments for dementia fall short of a cure. As such, identification of additional therapeutic strategies to enhance the health, function, and wellbeing of persons with dementia and their care partners is a priority of research and practice [2].

Physical activity and healthy eating are two interventions that support the health, function, and wellbeing of persons with dementia. Physical activity now forms a key part of many clinical recommendations for the management of dementia [3, 4]. Physical activity interventions improve fitness, balance, and mobility [5], reduce feelings of distress, and may decrease fall risk [6, 7]. Physical activity participation may also reduce cognitive decline among persons with dementia [8, 9], though not all studies agree [10]. Persons with dementia who take up physical activity have been shown to experience a 30% improvement in functional abilities [11] and their care partners report reduced care burden [12].

Healthy eating may also improve the wellbeing of persons with dementia. Healthy eating interventions promote ongoing health and function, regardless of the stage of dementia, especially since persons with dementia have greater malnutrition risk [13–17]. Persons with dementia are at increased risk of unintentional weight loss and malnutrition due to declines in appetite, changes in sensory perception, and challenges with acquiring, preparing, and consuming food [13–17]. Healthy eating strategies, dementia-inclusive mealtimes, and nutrition risk screening can be used to preserve weight and promote health [18–20]. Community programs to support physical activity, healthy eating, or wellbeing may yield additional benefits over individual-level interventions since they give persons with dementia an opportunity to socialize, develop meaningful relationships, and receive support and encouragement from program leaders and fellow participants [21, 22].

Affirmed by the United Nations Convention on the Rights of Persons with Disability [23], persons with dementia have a right to equal access to supports for their health and function through programs and services in their community [23]. Charters developed for and by people living with dementia similarly assert equal rights to supports health and independence [24]. Programs and services that support physical activity and healthy eating can reasonably be considered within these rights, yet structural and public stigma create ongoing barriers to the participation of persons with dementia [25, 26]. Community wellness programs and services are rarely designed with the intention of accommodating the needs of persons with dementia [25–28]. Even highly trained leaders of physical activity and healthy eating programs (i.e., exercise professionals and dietitians) report having little to no formalized education on how to understand and support the needs of persons with dementia in their programs and services [28–30]. This forces a reliance on ad hoc strategies to respond to challenges as they arise [28]. Creating a prepared workforce that has the necessary knowledge and skills to proactively plan for and meet the needs of persons with dementia is a key focus of human rights frameworks and dementia strategies [23, 24, 31, 32]. Towards this end, dementia-related training is essential for health care professionals and community service providers, including those who provide physical activity, healthy eating, and other wellness programs and services.

Development of such training should integrate the voices of persons with lived experience of dementia. Persons with dementia (along with other persons with disability) have the right to be actively involved in the decision-making for programs and services that will, or should, include them [23, 24]. Engagement of persons with dementia and their family members as co-creators in the development of training and resources can create more relevant and practical solutions [33, 34]. Co-development processes can also have broader impact on the team. Involving persons with dementia alongside multi-perspective partners during co-development processes helps overcome stigma, strengthens relationships among partners, and allows for integrated knowledge exchange [34–36].

The first published account of participatory or co-design research with persons with dementia only appeared in 2009 [37], though participatory and co-development activities likely preceded this by several years. The adoption of co-development approaches expanded considerably over the subsequent 15 years. Major organizations, including Alzheimer Europe, Alzheimer Scotland for example, engage working groups of persons with dementia to ensure projects, activities, and research align with the priorities, preferences, and needs of persons with dementia [38, 39].

The Dementia Resources for Eating, Activity, and Meaningful inclusion (DREAM) project had the overarching aim of expanding the number and quality of wellness programs and services available to persons with dementia, with a focus on physical activity and healthy eating. The purpose here is to detail the participatory process used to co-develop the DREAM tools and resources, describe the output of that process (the DREAM toolkit), and share lessons learned from the process. Due to COVID-19 related restrictions, in addition to the diverse geographies of our team, all activities were conducted virtually, yielding insights for the future of virtual participatory work with persons with dementia.

## DREAM participatory co-development process

The DREAM project was an evolution of our previous work, the Dementia-Inclusive Choices of Exercise (DICE) project (www.dementiaexercise.com) [40], which developed tools to give exercise providers the knowledge and skills to support persons with dementia with exercise. Through discussions among researchers, persons with dementia, and care partners, it was evident that there was an interest in expanding supports for the wellbeing of persons with dementia beyond the exercise setting. As a result, the DREAM Steering Team was assembled with the aim of improving and expanding supports for the wellbeing of persons with dementia in diverse settings (e.g., geographically, culturally) by increasing the number and quality of wellness programs and services that persons with dementia can access in their communities, focusing on physical activity (exercise and non-exercise) and healthy eating.

**Fig. 1:**
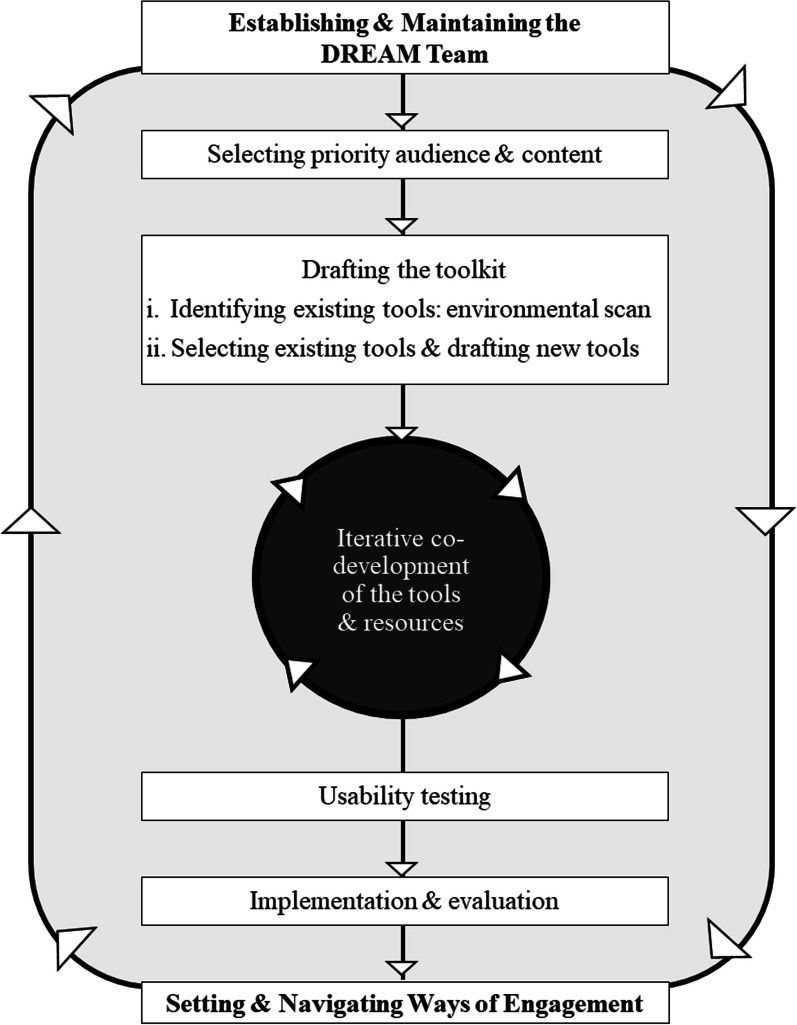
The DREAM co-design process

The DREAM project used a virtual, participatory process to finalize aims, set the project scope, and direct project activities. Our participatory process was guided by the principles and enablers of authentic partnerships, which were developed through and for participatory research with persons with dementia and care partners [33]. Authentic partnerships is an approach to including and valuing diverse perspectives, including those of persons with dementia and care partners, in decision-making [33]. The authentic partnership approach was developed over a decade of participatory work with persons with dementia and their families and outlines both guiding principles and enablers of authentic partnerships [33]. We followed the enablers and principles of authentic partnerships to create a space where members appreciated and respected each other, and where each person’s perspectives and experiences were valued and incorporated in the decision-making process [33, 34, 41]. In addition, the co-development process loosely aligned with the the steps of the Canadian Institutes of Health Research Knowledge to Action cycle, an iterative and dynamic process meant to capture knowledge creation through to its translation to practice and policy [42]. Specifically, our co-development process included the following steps: (1) Engaging and maintaining the DREAM Steering Team; (2) Setting and navigating ways of engagement; (3) Selecting the priority audience and content for the toolkit; (4) Drafting the content and format for the toolkit; (5) Iterative co-development of tools/resources; (5) Usability testing; and (6) Implementation and evaluation (Fig. 1).

## Engaging and maintaining the DREAM Steering Team

The DREAM Steering Team brought together diverse, multi-perspective partners: persons with dementia, family care partners, exercise professionals, dietitians, community and dementia service providers, and researchers from two institutions (University of Waterloo [UW] and University of Northern British Columbia [UNBC]). The DREAM Steering Team oversaw all project activities for the duration of the project (2021–2023), including refining project aims, outlining project scope, prioritizing audiences and key content areas, reflecting on and making decisions based on input from the co-development cycles, developing evaluation materials, and acting on evaluation results. Investigators and project staff, including a project coordinator, project manager, and student and non-student research assistants, carried out project activities based on the decisions of the DREAM Steering Team. Note that project staff were also part of the Steering Team, with the exception of student research assistants who were engaged for focused tasks.

DREAM Steering Team members were identified through several strategies. First, members of the DICE Steering Team were invited to the DREAM Steering Team. Several members agreed to be part of the DREAM Steering Team, including two persons with dementia, one community service provider, and three researchers. New members were then identified from the personal and professional networks of DREAM researchers and initial Steering Team members. Gaps in membership were identified early, and researchers connected with local organizations to identify additional members from underrepresented groups. We purposively engaged people of diverse ethno-cultural identities, linguistic groups, and geographical locations to ensure broad-ranging perspectives were included in our decision-making. We leveraged a patient engagement network (Patient Voices Network, patientvoicesbc.ca [43]) to identify additional persons with dementia and care partners. Persons with dementia and care partner members were compensated for their time.

The DREAM Steering Team included five persons with dementia, three care partners, three exercise professionals, four dietitians, and four community/dementia service providers alongside researchers, trainees, and project staff. Those individuals who were interested in the DREAM project but were unable to commit, or not interested in committing, to the DREAM Steering Team were invited to join for the more condensed iterative co-development period, which occurred over an intense 4-month period (summer 2021, described below).

The DREAM Steering Team membership evolved over the project, with some members reducing their level of engagement, changing how they engaged, or withdrawing completely due to personal or professional reasons. Where possible, we supported the changing abilities of persons with dementia to remain engaged—for example, by reviewing and discussing resources or processes individually by phone or Zoom (web conference platform) when group meetings became difficult to navigate. New members also joined the DREAM Steering Team during this process. Some were invited to ensure sufficiently diverse voices remained, and some individuals contacted the team to express interest after hearing about the project. For example, two persons with dementia joined the Steering Team in the second year of the project after participating in usability testing. They were involved in final decision making about adaptations to the DREAM toolkit, evaluation processes, and plans for dissemination.

## Setting and navigating ways of engagement

Early DREAM Steering Team meetings focused on *connecting* with other members and recognizing and *valuing the diverse perspectives and experiences* held among our Team, both enablers of authentic partnerships [33], and orientation to ethical issues related to co-development. We emphasized that Team members were all considered co-researchers within the Steering Team, with oversight for project processes and decision-making [44]. We recognized the diverse experiences and perspectives related to dementia among Team members and indicated that our goal was a safe space where all perspectives could be shared, respected, and valued, especially the lived experience of persons with dementia. We encouraged Team members to share personal experiences that they saw as relevant, and that these should be considered confidential within the Team. We recognized that sensitive topics may be discussed and emphasized that additional supports were available if needed, including 1-on-1 discussions to debrief on topics and processes. Members new to co-development processes were given a 1-on-1 orientation of the project and our approach prior to joining the full Steering Team meeting. Persons with dementia, in particular, were assured that their experiences and perspectives were considered central to all decision making. We made clear that any Team member could reduce the extent of their engagement, or withdraw completely, if needed or desired.

A secondary goal of early meetings was to establish processes for our engagement that facilitated *open communication* among our team. The Steering Team reflected on the appropriate frequency and duration of team meetings and decided to connect for 90-min monthly meetings via Zoom. We acknowledged that the frequency and duration of the meetings may vary over the duration of the project. The use of web-conference platforms for research, programs, and services had become quite common by 2021 due to uptake of virtual programs and meetings during the COVID-19 pandemic [45] and, as such, all team members had previously used Zoom to connect personally or professionally.

Agendas, relevant materials, and items for discussions and decision-making were shared via email approximately one week prior to each meeting. Meetings were used to reflect and discuss project-related issues and make decisions, often using breakout rooms to create manageable group sizes for discussions (approximately four to six people). A researcher (investigator, staff, or trainee) with experience working closely with persons with dementia facilitated the breakout room discussions with a second person taking notes. The facilitator purposely elicited the perspective of each member and sought to integrate perspectives and resolve differences in decision-making. Notes from individual breakout rooms were shared with the full team in the latter portion of the meeting. At times, Zoom polls were used to generate or confirm consensus decisions. Key differences were discussed and resolved either during the meeting, or by email and smaller meetings afterward. Steering Team members were encouraged to contact research staff or investigators if they had questions or concerns outside of meetings.

## Selecting the priority audience and content for the toolkit

The priority content of the DREAM toolkit was informed by prior research (including that from the DICE project). Barriers to participation in community wellness programs for persons with dementia were recognized within a socio-ecological model, existing at the levels of the individual with dementia, social relationships, and the community. Changes in cognitive, sensory, and physical abilities can be barriers to participation in physical activity and other community programs for persons with dementia [25, 27, 46]. Though there are fewer studies of the barriers to eating and mealtime programs [47–49], it is reasonable to expect changes common to dementia would similarly restrict participation. The impact of these changes on program participation, however, can be magnified by poor understanding of dementia and dementia-related stigma among program leaders and other attendees [27, 50]. Failure to plan for the inclusion of persons with dementia at a community- and societal-level creates barriers at multiple levels (e.g., low knowledge and adoption of dementia-inclusive practices among staff, failure to plan for dementia-friendly facilities and signage, and transportation with poor accessibility for persons with cognitive impairment) [25, 26, 51, 52].

Though changes are likely needed at multiple levels to create a truly inclusive community, intervention on the level of community service providers may be immediately actionable. Community service providers, such as physical activity providers and dieticians, report having little education or training regarding dementia [28, 51]. Physical activity providers, specifically, report having to address challenges on an ad hoc basis as they arise, searching for available resources to guide them in their actions [28]. We anticipated that community service providers who offer food- or meal-based programs, including dietitians [51], would similarly have little education and experience regarding dementia and varied experience with persons with dementia, resulting in low preparedness for inclusion of persons with dementia. Rights frameworks and dementia strategies emphasize a need for more and better trained providers to support the needs of persons with dementia in their communities [23, 24, 31, 52]. In the DICE project, we recognized that exercise providers could *enable* persons with dementia to overcome many individual, social, and environmental barriers to physical activity if the exercise providers understood dementia and had the skills to support persons with dementia in their programs and services [40]. Early meetings within the DREAM Steering Team discussed whether this approach—that is, enabling inclusion of persons with dementia by training the community service providers—was appropriate for the current project. Steering Team members confirmed that these groups rarely had education or training regarding dementia, in line with published research [28–30], which posed a barrier to participation of persons with dementia in community activities and wellness programs. Consequently, the DREAM Steering Team decided that educating community service providers who provide physical activity, food or meal, and wellness programs about the diversity of dementia and strategies to support persons with dementia was a priority of the project. We purposely embraced a broad definition of ‘community service providers’ to include both paid staff and volunteers who supported physical activity of any type (e.g., exercise, dance, walking, gardening), food, and/or mealtime programs in any community setting (e.g., community centres, cultural groups, religious institutions, businesses) and across diverse geographies (e.g., urban, rural, northern). We recognized that persons with dementia may choose to access supports in the settings that are most accessible or meaningful to them because of location, language, culture/ethnicity, or other factors.

Persons with dementia and care partners were identified as a secondary target audience. Though our actions would not provide wellness programs or services directly, our aim was to give persons with dementia and their families the knowledge and confidence needed to adopt physical activity and healthy eating and seek out additional community supports.

## Drafting the content and format of resources and tools

Having established the priority audience and aim, the DREAM Steering Team drafted, reflected on, discussed, and evolved the content areas and format for the DREAM tools. Where relevant, existing tools and resources (including those from DICE) were referenced. The DREAM Steering Team emphasized that information and training should be appropriate to various levels of physical activity and nutrition expertise. The Steering Team also recognized a need to represent diverse approaches to physical activity and eating patterns to accommodate different cultural traditions and personal preferences, and to consider accessibility of facilities and food (especially in rural and northern communities). Though nutrition risk and fall risk would not be a focus of the DREAM toolkit, the Steering Team agreed that Additional file 1 resources would be useful so they could be consulted or shared if issues arose. The in-depth discussions among the DREAM Steering Team resulted in a focus on seven content areas (Table 1).

When considering the best format for delivering content and messages, the DREAM Steering Team thought that including resources of diverse formats (e.g., videos and case studies sharing the lived experience of persons with dementia, learning modules with a certificate of completion at the end, and handouts that could be shared with persons with dementia and their families (on-line and in print) would be most effective. A summary of the purpose and importance of each resource and integration of practical strategies to promote inclusion (e.g., case studies) were emphasized as important.﻿

**Table 1:** Mapping of existing tools identified through the environmental scan to priority content areas

Content area	Existing tools available		Areas where new/adapted tool(s) are needed
	To use as is	Adaptation needed	
Understanding the diversity of dementia	X	X	Adapted tool to share interests, abilities, history of person with dementia & supports needed
Rights of persons with dementia	X		
Core dementia-inclusive principles	X	X	
Benefits of physical activity for persons with dementia	X		
Dementia-inclusive strategies for physical activity	X		Simple resources to share easy strategies to be active, screening for safety for exercise
Benefits of healthy eating for persons with dementia			Up-to-date resources about healthy eating and dementia, strategies to make healthy (and accessible) food choices
Dementia-inclusive strategies for shared mealtimes			Resources to share strategies for overcome mealtime challenges and share preferences/needs of person with dementia

Note that there was often more than one tool identified for each area so there may be marks in more than one column

### Identifying existing resources: environmental scan

In parallel to the finalization of content areas, an environmental scan of leading dementia, physical activity, and healthy eating websites was conducted (January–April 2021). The aim was to identify high-quality existing resources related to dementia, dementia-inclusive practices, and dementia in relation to physical activity or healthy eating that may be suitable for consideration by the DREAM Steering Team for inclusion in the DREAM toolkit. Examples of websites searched include Alzheimer Society/Association, World Health Organization, Government of Canada, Public Health Agency of Canada, as well as various physical activity and nutrition associations. Resources related to guidelines, recommendations, education, training, interventions, programs, and services were considered for inclusion. However, resources focusing on preventing cognitive impairment or dementia, or that were experimental in nature, were excluded.

During the environmental scan, 68 websites were searched and 215 resources (62 on dementia inclusion, 56 on physical activity, and 97 on healthy eating and mealtimes) were identified. Three DREAM Steering Team researchers reviewed and rated the resources based on clarity, relevance, accuracy, readability, and visual appeal using an assessment tool derived from valid and reliable measures of these factors (that is, the DISCERN tool and the Checklist for Evaluating Learning Materials) [53, 54]. Additional information was noted including: (1) available languages; (2) relevance to rural/remote geographies; (3) involvement of persons with dementia, care partners, and/or service providers in the resource development; (4) evaluation of effectiveness; (5) content gaps; and (6) uniqueness. Researchers made note if specific elements of the resource were well done (e.g., style, readability) and/or whether the resource could be improved through adaptation. Through rankings and discussion, 31 resources were considered sufficiently high-quality and relevant to be considered further by the DREAM Steering Team for inclusion in the iterative co-development process.

### Selecting existing tools and drafting new tools

The DREAM Steering Team reviewed and reflected on the identified high-quality resources to determine which could potentially be used as-is or adapted, and where gaps in content existed (detailed in Table 1). For example, no nutrition or mealtime resources were considered appropriate as is. Other resources meant to increase understanding of dementia or dementia-inclusive principles were identified but were thought to need considerable revisions. For gap areas, project staff drafted high-level content outlines based on best-evidence to meet gaps, which were circulated to Steering Team members. During the meetings, Steering Team members shared their thoughts and feedback in breakout room discussions and full-team summary discussions. Dialogue and decisions were documented by research staff. In some cases, there were existing Canadian tools but higher-quality international tools. In these cases, the Steering Team generally preferred that new or updated Canadian tools be created with the ‘higher-quality’ elements integrated, which were drafted accordingly. The Steering Team preferred Canadian tools for diverse reasons, including differences in language, terminology, and health and social care contexts.

## Iterative co-development of tools and resources

**Fig. 2:**
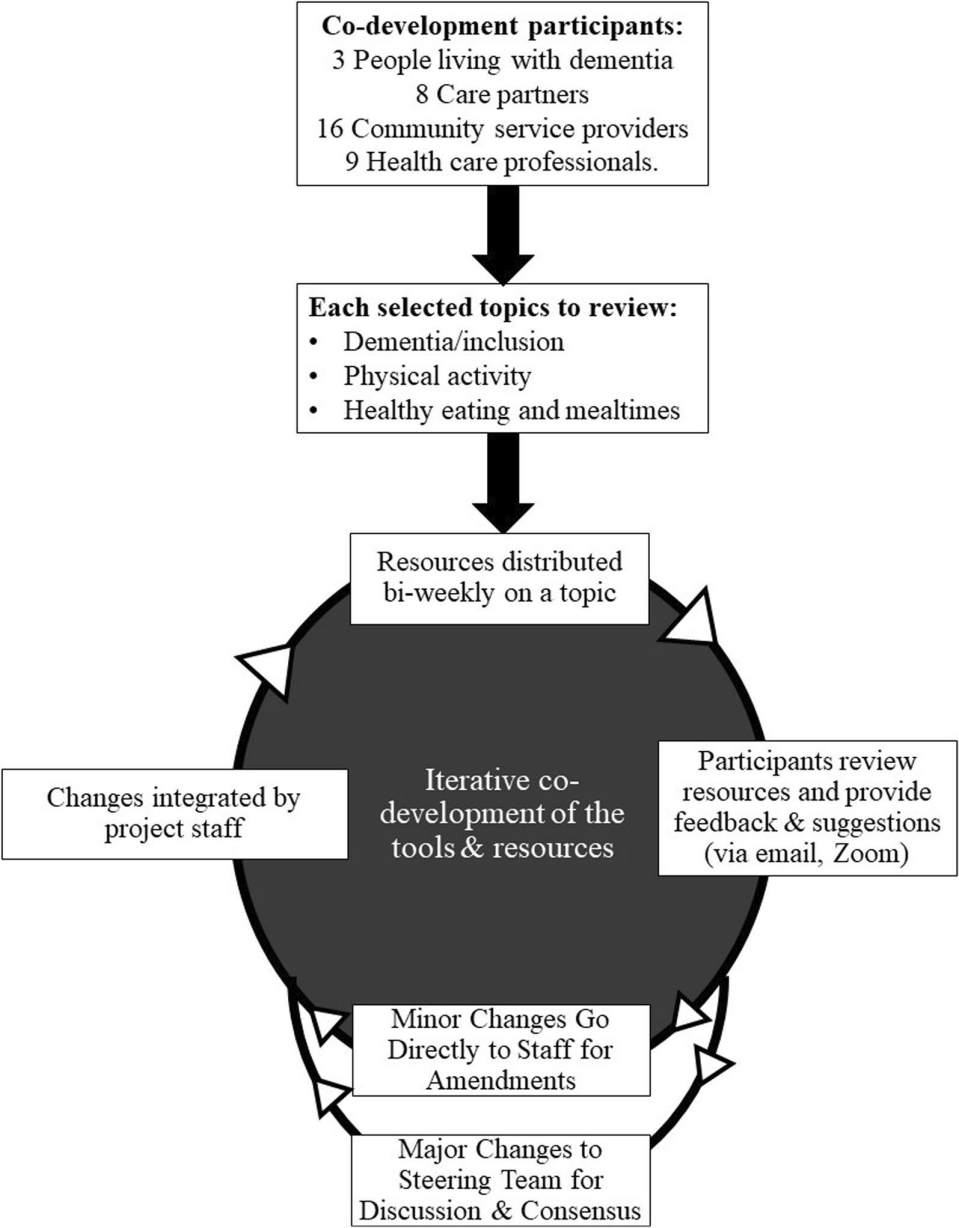
Iterative co-development process to refine DREAM toolkit

The DREAM Steering Team, along with an additional 28 multi-perspective partners were engaged for iterative rounds of reflection, feedback, and adaptation of identified existing and new tools and resources. Participants in this iterative co-development phase were asked to provide suggestions, feedback, and revisions regarding content, format, and design of each tool, whether new or existing (detailed in Fig. 2). We emphasized that the purpose of the iterative, co-development cycles was to review and critically reflect on the tools and resources identified or drafted so far, noting that these should only be considered a starting point.

A total of 36 people (3 persons with dementia, 8 care partners, 16 community service providers, and 9 health care professionals) participated in the iterative cycles of co-development of the tools and resources from April to September 2021. More than half (64.3%) of participants in this phase were from Ontario (Canada) with the remainder residing in British Columbia (Canada). We aimed to engage diverse voices within the co-development process and partially achieved this aim. Eight participants identified as a newcomer to Canada and 10 identified as from minority ethno-cultural groups (that is, not Caucasian/White). Though English was the predominant first language (85%), 35% also spoke another language, including Arabic, Cantonese, Creole, French, Gujrati, Hindi, Italian, Mandarin, Urdu, and Yoruba. Half of the participants identified as from a rural (8 people) or northern (10 people) geography, enabling them to reflect and provide input based on access issues related to food, facilities, and expertise that is common in these geographies.

Each participant indicated the topic(s) (dementia/inclusion, physical activity, and/or healthy eating and shared mealtimes) for which they wanted to review resources, based on their interests and expertise. Research staff sent weekly emails with a subset (usually two to three) of the existing and draft new resources for review, batched by content area. When requested, the resources were also sent by mail. Participants had 1–2 weeks to review each batch of resources and provide feedback. To accommodate the diverse abilities and preferences of co-development participants, individuals could share feedback in a variety of ways including tracked changes in the documents, sharing comments via email, and discussions via telephone or Zoom. Participants suggested minor revisions (e.g., correcting errors, simplifying wording), major revisions (e.g., adding case studies, links to other resources), and/or additions in scope (e.g., Additional file 1 content regarding care partner stress). Research staff recorded and integrated minor changes as feedback was received. Suggested major changes and additions in scope were shared with the DREAM Steering Team for reflection and discussion prior to a final decision on implementation. Zoom polls were used for some decisions to ensure we reached consensus on a path forward. Additional meetings or email communications were used, as needed, to resolve conflicting perspectives (e.g., for dietary recommendations for persons with dementia). Revised resources were then recirculated to the DREAM co-design participants in July 2021, with emails going out each week. Each email specified whether minor or major revisions were made to the resources so that co-development participants could prioritize their time accordingly. Where needed, a third round of revised resources was sent during August 2021, which included a few resources that had undergone larger changes. By the fourth round, all co-development participants considered the resources ready for usability testing.

At the end of the co-development phase, the DREAM toolkit included a website, seven learning modules with an accompanying learning manual, six videos, 12 new resources, and 36 existing resources (31 from environmental scan and five identified after the scan), detailed in Table 2. The DREAM website (www.dementiawellness.ca) includes a section targeted to community service providers and a section targeted to persons with dementia (and their care partners), with group-relevant resources. Key content is available through learning modules for community service providers or webpages for persons with dementia and family members. An accompanying printable learning manual for each group was created, summarizing key information. Relevant videos and resources are embedded within the learning modules and website sections, and available independently on resource pages and a YouTube channel (www.youtube.com/@dementiawellness).

**Table 2:** A summary of the DREAM Toolkit

Tool or resource	Topic/title
Seven learning modules for community service providers	Diversity of dementia
	Rights of persons with dementia
	Core dementia-inclusive principles
	Benefits of physical activity for persons with dementia
	Dementia-inclusive strategies for physical activity
	Benefits of healthy eating for persons with dementia
	Dementia-inclusive strategies for mealtimes
Learning manual: community service providers	Topics aligned with learning modules
Learning manual: persons with dementia and care partners	Topics aligned with learning modules
Six videos	Title: *I have dementia but I’m still me*
	Title: *Advice for living well with dementia*
	Title: *Staying active helps me live well with dementia*
	Title: *Dementia inclusive physical activity: A little support goes a long way*
	Title: *Healthy eating helps me live well with dementia*
	Title: *Eating together brings me joy with dementia*
12 new resources	Diversity of dementia & rights: 1 new resource
	Physical activity: 2 new resources, 2 new wallet cards
	Healthy eating: 5 new resources, 2 new wallet cards
36 existing resources	Diversity of dementia & rights: 17 resources
	Physical activity: 6 resources
	Healthy eating: 13 resources

## Usability testing

After the co-development process, we conducted usability testing of the DREAM website and embedded tools and resources with persons with dementia, care partners, and community service providers who were not members of the DREAM Steering Team or involved in the co-development process. Purposive sampling was used to recruit participants representing each participant category by sharing project-related emails with partner organizations and networks (e.g., YMCA, local Alzheimer’s Society, Dementia Advocacy Canada). The usability study was approved by the University of Waterloo Office of Research Ethics (ORE #43097) and the University of Northern British Columbias Research Ethics Board (REB #E2022.0203.005.00). All participants provided verbal informed consent.

Using a brief questionnaire, participants reported demographic information and, in the case of community service providers, descriptions of their training and practice. Participants were then asked to share their computer screen using Zoom and complete a series of usability tasks on the DREAM website, describing their thoughts as they completed each step. A research assistant noted whether participants were able to complete the tasks, time needed to complete tasks, challenges experienced, and comments about the tools and resources. After the Zoom session, participants were asked to review the rest of the DREAM toolkit, including the website, learning modules (community service providers only), and other resources (all participants). Participants recorded their thoughts and experiences in a reflection diary, noting how easy it was to use the resource, what they liked, what they would change, and whether they would share the tool with others. Community service providers were asked whether they thought that DREAM learning modules would improve their ability to meet the needs of persons with dementia in their programming, and if they felt the DREAM website shared information and resources in an accessible way. After this review period, participants completed a semi-structured interview to reflect on their experience with the DREAM tools. A $50 e-gift card honorarium was provided to participants in recognition of their time and contributions.

Seven community service providers (mean age 37 years), five persons with dementia (mean age 71 years), and six care partners (mean age 56 years) participated in the usability testing. All the community service providers and care partners, and 40% of persons with dementia were women. Half of the community service providers offered physical activity programs and services, and the other half offered food or meal services. Based on the usability tasks, participants from all groups generally found the website easy to use and liked design elements (e.g., fonts, colours). However, some participants experienced challenges with navigating the website and learning modules, or disliked scrolling down on long webpages.

Like the iterative feedback stage, minor changes were made immediately by research staff. Minor changes included fixes or minor changes to spelling, grammar, and format that were unlikely to be objectionable. Major changes were reflected on and discussed within the DREAM Steering Team prior to implementation to ensure they were feasible and within scope. The decision on whether changes required discussion by the Steering Team was made by staff in consultation with investigators. If there was any doubt, the change was brought to the Steering Team. Across all groups, participants said the videos and graphics were informative and engaging. One community service provider participant described the relevance of learning modules: “I thought the learning modules were very relevant. Like, all the things that we talked about in the different modules was like, I thought oh, I can use these in real life interactions and real-life experiences, which is great.” A care partner participant reported that “if you went through all of the topics, you got a good mix of resources that would cover everything that you would need, while going through this journey.” A person with dementia also spoke about the long-term impact of DREAM: “I think it’s an amazing site that’s going to do–it’s going to bring so much–the word I’m looking for is it’s going to bring value to our lives, for so many reasons. The number one reason is that it is being done in the first place, which means we have some importance in the system”.

## Implementation and evaluation

### Process evaluation

At the end of the iterative co-development phase, people were not on the DREAM Steering Team but who participated in the co-development (*n* = 28) were invited to complete a survey on how this process aligned with the guiding principles and enablers for authentic partnerships. We used a brief survey, developed by our team to evaluate another participatory project, with minor amendments (Additional file 1). All participants provided informed consent online (University of Waterloo Office of Research Ethics # 42427). Respondents were asked to report their role on the project; some roles were collapsed to a single category to reduce identification of individuals (e.g., persons with dementia and care partners were a single category; community service providers were inclusive of exercise, food or meal, and dementia service providers). Respondents reported whether the DREAM co-development process aligned with the principles of authentic partnership on a 5-point Likert scale from strongly agree to strongly disagree. Using the same scale, the respondents reported on our communication, their level of engagement in the co-development process, and the use of technology for our project. The survey was administered online using Qualtrics survey software (Qualtrics XM). Participants in the DREAM iterative co-development phase were invited to the survey by email. Reminder emails about survey participation were sent to participants 2 weeks after the initial invitation. In addition, a final virtual meeting was convened to thank the DREAM co-development participants for their contributions and to reflect on the virtual co-design process. Poignant comments were recorded for reflection.

Fourteen of the 28 co-development participants responded to the survey. Service providers (57.1%) were the most representative group. All respondents either strongly agreed or agreed that they felt respected, safe to share their opinions, and valued by others during the DREAM resource co-design process. Furthermore, all respondents thought that other team members shared valuable experiences, knowledge, and perspectives, and that the individuals involved in the project had the appropriate expertise. Over 80% of respondents agreed that they were able to meaningfully contribute to the DREAM resource co-development process, and that the DREAM project helped them learn something new about dementia and/or strategies to improve wellbeing. The vast majority of participants agreed that the DREAM project would have a positive impact on persons with dementia and care partners (93.4% and 93.3%, respectively). Results are detailed in Table 3.

**Table 3:** Co-development participant respondents (n = 14) ratings of how our process aligned with the principles and enablers of authentic partnerships (Likert scale strongly disagree = 1, strongly agree = 5)

	Strongly disagree	Disagree	Neither agree nor disagree	Agree	Strongly agree	Average (/5)
I felt respected by other DREAM co-design participants	0 (0%)	0 (0%)	0 (0%)	3 (21%)	11 (79%)	4.8
I felt safe to share my opinions during the DREAM co-design project	0 (0%)	0 (0%)	0 (0%)	2 (14%)	12 (86%)	4.9
I felt that my opinions and perspectives were valued for their contribution to the DREAM co-design process	0 (0%)	0 (0%)	0 (0%)	4 (29%)	10 (71%)	4.7
I felt the other DREAM co-design participants shared valuable experiences, knowledge, and perspectives	0 (0%)	0 (0%)	0 (0%)	2 (14%)	12 (86%)	4.9
I felt that I was able to make valuable contributions to the DREAM co-design process	0 (0%)	0 (0%)	3 (21%)	3 (21%)	8 (57%)	4.4
I felt that the types of people included in the DREAM co-design process had all the appropriate expertise	0 (0%)	0 (0%)	0 (0%)	5 (36%)	9 (64%)	4.6
I felt that participation in the DREAM co-design process helped me learn something new about dementia or strategies to improve well-being of people living with dementia	0 (0%)	1 (7%)	1 (7%)	6 (43%)	6 (43%)	4.2
I felt connected to the whole DREAM co-design group	0 (0%)	0 (0%)	5 (36%)	5 (36%)	4 (29%)	3.9

### Toolkit evaluation

A full evaluation of the DREAM toolkit is planned to understand the impact of the toolkit among diverse persons with dementia, care partners, and community service providers. Evaluation tools and processes were drafted by DREAM investigators, and then brought to the DREAM Steering Team for reflection, feedback, and revised accordingly. Evaluation tools were piloted with DREAM members living with dementia to gather feedback on the acceptability and feasibility of the questions. Assessment questions or tools that were confusing were altered or replaced. Results of the DREAM toolkit evaluation will be disseminated elsewhere.

## Reflections and discussion

We used a virtual participatory process to co-develop the DREAM toolkit with an aim to the increase the number and quality of supports for the physical activity, healthy eating, and wellness of persons with dementia. Our project was overseen by the DREAM Steering Team, which included multi-perspective partners including persons with dementia, care partners, dementia and community service providers, and health care professionals. Additional partners were engaged within the 4-month iterative co-development period. A brief evaluation of this co-development process indicated that participants believed that the process aligned well with the principles and enablers of authentic partnership, despite being conducted over an accelerated period and entirely on-line. Optimistically, these results suggest that meaningful collaboration where participants feel valued and respected is achievable even with minimal face-to-face contact. Initial reflections from participants in our usability study suggest the co-developed DREAM toolkit may be an accessible, relevant, and impactful way to train community service providers to meet the needs of persons with dementia in their physical activity, healthy eating, shared mealtimes, and wellness programs and services.

Participatory co-development processes are inherently influenced by the team members involved, as well as their personal histories. In this project, we purposively engaged a diverse, multi-perspective team that included the lived experience of dementia (persons with dementia and care partners), and people from diverse geographies (urban, rural, and northern) and ethno-cultural groups. One of our two lead sites was in a northern Canadian city (Prince George, BC), which is a hub for health care and community services for northern urban and rural communities. As a result, considerations important to these geographies (e.g., harsh weather and relatively low availability of facilities, services, and trained professionals) were integrated in all decision-making. In addition, we included people with diverse ethno-cultural identities, where 28% of co-development participants described themselves as having a minority ethno-cultural identity (i.e., non-white), similar to the general Canadian population (25%) [55]. Nonetheless, a small number of individuals cannot represent entire cultural groups so ongoing and broader engagement is required. Furthermore, the co-development participants all spoke English, which enabled deep discussion but likely biases their preferences in relation to physical activity and eating.

Time and patience are needed to develop strong relationships, share perspectives, and resolve differences across diverse groups, especially among those who experience stigma such as Indigenous Peoples and people with dementia with numerous ethno-cultural backgrounds [56]. Careful discussion and consensus building was needed when perspectives directly conflicted. One prominent example was the preferred terminology for dementia. Some participants preferred avoiding the term as it was heavily stigmatized in their culture, others felt it was important to use dementia as a step to overcoming stigma, while others indicated that their language had no word for dementia (e.g., some Indigenous languages). Terminology for dementia was a topic of careful discussion within the Steering team. In consultation with the Team and other co-development participants, we decided to use the term ‘dementia’ but acknowledge the associated stigma and that terminology and perspectives on dementia vary broadly across cultures early in training materials. To integrate the ‘ideal’ language and format for each culture, unique resources for each community may be ideal though resource intensive.

Due to the timing of the project alongside COVID-19 outbreaks and public health restrictions on gatherings and travel, all project processes occurred virtually. We did not identify any published studies that engaged persons with dementia in entirely virtual co-development processes. However, groups have conducted participatory research with persons with dementia over the last two decades, with the first published in 2009 [37] but other work preceding this by several years. For example, the Murray Alzheimer Research and Education Program in Canada started its participatory work with persons with dementia in 2002, which led to the development of the authentic partnership approach followed here [33]. During the DREAM project, we elicited and valued the perspectives of our members with dementia, which were considered foundational to all decisions. We used virtual meeting and features (e.g., breakout rooms, polls), email, and telephone to engage and support their contributions. As the abilities of our DREAM Steering Team members changed, we found new ways to support them when needed. For example, one individual who started to feel overwhelmed in the large group, virtual meetings instead provided input on decisions by meeting with a key staff member or investigator one-on-one over Zoom or by providing thoughts via email, attending larger Zoom meetings when having a ‘good’ day. Finding effective ways to include persons with dementia in participatory research as their symptoms become more progressed is needed and may require supporting the inclusion of their voices in alternative ways, such as one-on-one engagements using other forms of communication (e.g., photo-, story, and other arts-based methods) [33].

Guidance for virtual, health-oriented co-design describes accessibility as a key consideration for virtual co-design [57]. All our partners, including those with dementia, had used Zoom prior to the project. This was not uncommon by winter 2021 as COVID-19-related public health restrictions had accelerated the move to virtual programs and services, including those for persons with dementia (e.g., Alzheimer Society programs), as well as the adoption of technologies by older adults (where 88% of older Canadians reported using the internet daily in 2020) [45]. Virtual meetings allowed us to engage geographically diverse team members (urban, rural, remote, northern). Furthermore, virtual meetings also allowed us to be more efficient in time, efforts, and resources needed for transportation, which may have supported the engagement of a more diverse ethno-cultural team. However, the cost of technologies and the reach of internet services remain barriers to full accessibility. Approximately 15% of Canadians have insufficient data speeds for virtual meetings [58], though major government initiatives aim to provide high-speed internet access to 95% of the country by 2026 [59].

We identified only one published study that focused on co-design or participatory research done remotely with persons with dementia, which examined telepresence robots as a facilitator to remote engagement in long-term care and described various strengths and challenges [60]. In addition, recent publications describe virtual co-design with other groups using unique combinations of virtual meetings and/or digital participatory tools (e.g., [57, 61–63]). Here, we used relatively simple and commonly used technologies (Zoom web conferencing and polls, email, and telephone) to engage our team to minimize barriers to participation. Zoom was widely used by the Alzheimer Societies in Canada by 2021 and was perceived as the most dementia-friendly web-conference platform. Making decisions within a single platform avoided the need for Team members, including those living with dementia, to learn how to use other collaboration platforms.

Guidance for virtual co-design processes emphasize the importance of collaboration, communication, and facilitation [57]. In this project, we used initial meetings to *connect* members and *commit* to set project aims and scope (both enablers of authentic partnerships). Emails were used to communicate project activities and introduce key questions and materials for decision-making. Most often, virtual meetings were used to make collective decisions. Meeting facilitators had substantial experience engaging with persons with dementia and multi-perspective groups. We used breakout rooms for small group discussions, where facilitators elicited and valued each person’s perspective. Careful planning of breakout room composition, and re-balancing when needed, resulted in the active engagement of almost all Team members in discussions. Sometimes, small group breakout room discussions took varied directions, which could be challenging for consensus building. Over the course of the project, we learned that a two-stage, anonymous polling process was useful for making final decisions after discussion. The first poll asked for the favoured solution. If there was a sufficiently favored option, we then used a second poll to see if all members favoured moving forward with the consensus decision or whether more discussion was needed.

We engaged the multi-perspective DREAM Steering Team and additional partners in authentic partnership [33], as confirmed by a participant survey. Meaningful partnership with interested parties can create more meaningful and relevant solutions. Indeed, the co-developed learning modules, videos, and resources were considered relevant and useful by usability study participants. It was perhaps surprising that co-development participants, who were engaged almost entirely by email with start-up and wrap up Zoom meetings, still felt the co-development process aligned with the principles and enablers of authentic partnerships. We posit seeing their feedback integrated into the tools and resources over the iterative process demonstrated that their input was valued.

The DREAM toolkit fills a key knowledge translation gap in educational resources about dementia for community service providers, specifically targeting those who provide physical activity, eating, and mealtime programs and services who previously reported a poor understanding of dementia [28–30]. While general dementia-friendly training may be useful, the DREAM toolkit specifically addresses the rights to, benefits of, and practices for physical activity and eating programs for persons with dementia. By providing community service providers with education to understand dementia and gain strategies to support persons with dementia in their programs and services, we expect that trained community service providers will be equipped to enable the participation of persons with dementia in community programs and services that support their health and wellbeing in the locations close to home, a key right of persons with disability [23]. Furthermore, by recognizing and supporting the rights of persons with dementia to equal participation in their communities, community service providers support their personhood and social citizenship [64, 65]. Access to and participation in physical activity and food-based interventions, in particular, may support the health and functional independence of persons with dementia [4].

### Limitations

Despite an effective co-development process that aligned with authentic partnership, our project has limitations that restrict the generalizability of our approach. First, our persons with dementia were willing to self-identify as such. By nature, those unwilling to disclose their dementia diagnosis were excluded and may reflect a different group with greater experiences of stigma. In addition, all team members had access to computers and were able to use Zoom. This allowed us to engage people across diverse geographies. Yet people without adequate technologies were not able to participate. Also, we engaged persons with dementia across mild to moderate stages, adapting methods for engaging individuals as their needs changed. However, even those in moderate stages had relatively well-preserved language abilities, which facilitated ongoing engagement. Additional strategies may be needed to further support those with early language impairment, or severe sensory impairments, which make virtual communication more difficult.

## Conclusion

By adhering to the principles and enablers of authentic partnerships, we were able to engage multi-perspective partners within a participatory co-development process where each individual was valued and able to contribute, even when using entirely virtual engagement. Usability testing suggests that the DREAM resources created are relevant, acceptable, and may have a positive impact on the inclusion of persons with dementia in wellness programs and services. Our co-developed resources fill identified gaps in knowledge translation materials to educate community service providers regarding dementia and dementia-inclusive services, with the end goal of improving the health and wellbeing of persons with dementia and their care partners by creating inclusive programs and services in their communities.

### Supplementary Information

**Additional file 1.** Survey to assess alignment with principles and enablers of authentic partnership.

## Data Availability

The datasets generated and/or analysed during the current study are not publicly available as they reflected steps within a participatory research process but are available from the corresponding author on reasonable request.
