# Supplementary material for: Dementia resources for eating, activity, and meaningful inclusion (DREAM) toolkit co-development: process, output, and lessons learned
Source: Res Involv Engagem. 2023 Sep 30;9:87. doi: 10.1186/s40900-023-00497-4 (PMC10542247; doi:10.1186/s40900-023-00497-4)
Supplement: Supplementary file 1 — Additional file 1. Survey to assess alignment with principles and enablers of authentic partnership. [file 40900_2023_497_MOESM1_ESM.pdf]

DREAM Co-Design Follow-Up Survey

DREAM Co-design Follow-up Survey

DREAM Co-design team role

1. What group did you represent in the meetings?
   - ☐ Person living with dementia or family/friend care partner to a person living with dementia
   - ☐ Community service provider
   - ☐ Healthcare provider
   - ☐ Other, please specify: ______________________________________

DREAM Co-design team and processes

We would like to get your feedback on your experiences participating in the DREAM co- design process, where you helped to co-create a program/resources to support living well with dementia. Please indicate the extent to which you agree with the following statements about your experience with the DREAM co-design process.

2. I felt respected by other DREAM co-design participants.

   - ☐ Strongly agree
   - ☐ Agree
   - ☐ Neither agree nor disagree
   - ☐ Disagree
   - ☐ Strongly disagree

3. I felt safe to share my opinions during the DREAM co-design meetings.

   - ☐ Strongly agree
   - ☐ Agree
   - ☐ Neither agree nor disagree
   - ☐ Disagree
   - ☐ Strongly disagree

4. I felt that my opinions and perspectives were valued for their contribution to the DREAM co-design process.

   - ☐ Strongly agree
   - ☐ Agree
   - ☐ Neither agree nor disagree
   - ☐ Disagree
   - ☐ Strongly disagree

DREAM Co-Design Follow-Up Survey

5. I felt the other DREAM co-design participants shared valuable experiences, knowledge, and perspectives.

    - ☐ Strongly agree
    - ☐ Agree
    - ☐ Neither agree nor disagree
    - ☐ Disagree
    - ☐ Strongly disagree

6. I felt that I was able to make valuable contributions to the DREAM co-design process.

    - ☐ Strongly agree
    - ☐ Agree
    - ☐ Neither agree nor disagree
    - ☐ Disagree
    - ☐ Strongly disagree

7. I felt that the types of people included in the DREAM co-design process had all the appropriate expertise.

    - ☐ Strongly agree
    - ☐ Agree
    - ☐ Neither agree nor disagree
    - ☐ Disagree
    - ☐ Strongly disagree

8. I felt that participation in the DREAM co-design process helped me learn something new about dementia and / or strategies to improve well-being of people living with dementia.

    - ☐ Strongly agree
    - ☐ Agree
    - ☐ Neither agree nor disagree
    - ☐ Disagree
    - ☐ Strongly disagree

9. I felt connected to others in my smaller breakout group.

    - ☐ Strongly agree
    - ☐ Agree
    - ☐ Neither agree nor disagree
    - ☐ Disagree
    - ☐ Strongly disagree

DREAM Co-Design Follow-Up Survey

10. I felt connected to the whole DREAM co-design group.

- ☐ Strongly agree
- ☐ Agree
- ☐ Neither agree nor disagree
- ☐ Disagree
- ☐ Strongly disagree

11. I felt it was easy to effectively communicate with other participants during the DREAM co-design process.

- ☐ Strongly agree
- ☐ Agree
- ☐ Neither agree nor disagree
- ☐ Disagree
- ☐ Strongly disagree

12. I felt it was easy to understand other people's ideas during the DREAM co-design process.

- ☐ Strongly agree
- ☐ Agree
- ☐ Neither agree nor disagree
- ☐ Disagree
- ☐ Strongly disagree

13. After the DREAM co-design process I thought about the ideas and perspectives shared during the process.

- ☐ Strongly agree
- ☐ Agree
- ☐ Neither agree nor disagree
- ☐ Disagree
- ☐ Strongly disagree

14. Before participating in the DREAM co-design process, I was comfortable using video-conferencing technology.

- ☐ Strongly agree
- ☐ Agree
- ☐ Neither agree nor disagree
- ☐ Disagree
- ☐ Strongly disagree

15. During the DREAM co-design process, I found that the video-conferencing technology was easy to use.

- ☐ Strongly agree
- ☐ Agree
- ☐ Neither agree nor disagree
- ☐ Disagree
- ☐ Strongly disagree

16. During the DREAM co-design process, I found that the video-conferencing technology was reliable

- ☐ Strongly agree
- ☐ Agree
- ☐ Neither agree nor disagree
- ☐ Disagree
- ☐ Strongly disagree

DREAM Program

---

17. If implemented, I think the DREAM toolkit will have a positive impact on people living with dementia.

- ☐ Strongly agree
- ☐ Agree
- ☐ Neither agree nor disagree
- ☐ Disagree
- ☐ Strongly disagree

18. If implemented, I think the DREAM toolkit will have a positive impact on the family / friend care partners of people living with dementia.

- ☐ Strongly agree
- ☐ Agree
- ☐ Neither agree nor disagree
- ☐ Disagree
- ☐ Strongly disagree

DREAM Co-Design Follow-Up Survey

Is there any feedback you would like to share about your experience with the DREAM co-design to help improve the process in the future?

____

____

____

____

____
